# Genetics of melanoma

**DOI:** 10.3389/fgene.2012.00330

**Published:** 2013-01-25

**Authors:** Janet Wangari-Talbot, Suzie Chen

**Affiliations:** Susan Lehman Cullman Laboratory for Cancer Research, Ernest Mario School of Pharmacy, Rutgers, The State University of New JerseyPiscataway, NJ, USA

**Keywords:** melanoma, MAPK, PI3K/AKT, *GRM3*, *PREX2*, *BRAF*, *RAC1*

## Abstract

Genomic variation is a trend observed in various human diseases including cancer. Genetic studies have set out to understand how and why these variations result in cancer, why some populations are pre-disposed to the disease, and also how genetics affect drug responses. The melanoma incidence has been increasing at an alarming rate worldwide. The burden posed by melanoma has made it a necessity to understand the fundamental signaling pathways involved in this deadly disease. Signaling cascades such as mitogen-activated protein kinase and PI3K/AKT have been shown to be crucial in the regulation of processes that are commonly dysregulated during cancer development such as aberrant proliferation, loss of cell cycle control, impaired apoptosis, and altered drug metabolism. Understanding how these and other oncogenic pathways are regulated has been integral in our challenge to develop potent anti-melanoma drugs. With advances in technology and especially in next generation sequencing, we have been able to explore melanoma genomes and exomes leading to the identification of previously unknown genes with functions in melanomagenesis such as *GRIN2A* and *PREX2*. The therapeutic potential of these novel candidate genes is actively being pursued with some presenting as druggable targets while others serve as indicators of therapeutic responses. In addition, the analysis of the mutational signatures of melanoma tumors continues to cement the causative role of UV exposure in melanoma pathogenesis. It has become distinctly clear that melanomas from sun-exposed skin areas have distinct mutational signatures including C to T transitions indicative of UV-induced damage. It is thus necessary to continue spreading awareness on how to decrease the risk factors of developing the disease while at the same time working for a cure. Given the large amount of information gained from these sequencing studies, it is likely that in the future, treatment of melanoma will follow a highly personalized route that takes into account the differential mutational signatures of each individual’s cancer.

## INTRODUCTION

The incidence of melanoma has been rising at an alarming rate in both men and women especially in the Caucasian population (Purdue et al., 2008). According to the American Cancer Society, the lifetime risk of developing melanoma currently stands at 2% in whites, 0.1% in blacks, and 0.5% in Hispanics (American Cancer Society, 2012). It has been proposed that this increase is a result of correction in underreporting through the Surveillance, Epidemiology, and End Results (SEER) program (Cockburn et al., 2008), increased surveillance and diagnosis (Jemal et al., 2001), and an increase in risky behaviors such as indoor tanning (Armstrong and Kricker, 2001; Lazovich et al., 2010). Regardless of the cause of rise in incidence, an increase in survival after a diagnosis of metastatic melanoma has also been noted with the development of new therapies. Targeted therapies such as vemurafenib (Chapman et al., 2011) have emerged from advances in genetic profiling of molecular targets and it is expected that as new targets are identified, novel therapies will continue to emerge. Three key molecular pathways have been found to be highly deregulated in melanoma: mitogen-activated protein kinase (MAPK), as a result of mutations in *RAS*, *RAF*, and *KIT*; PI3K/AKT, as a consequence of mutations in *RAS*, mutations or loss of *PTEN* (phosphatase and tensin homolog) and dysregulated expression of *AKT*, and p16INK4A due to mutations in *CDKN2A*, *ARF*, and *p53*. Various strategies of targeting melanoma have emerged based on the information gained from analyses of these pathways with varying success. Molecular genome screens of tumor samples have been instrumental in identifying novel targets in melanoma. In this review, we will discuss the aforementioned pathways as well as novel emerging targets identified in large-scale tumor genome profiling studies.

## MITOGEN-ACTIVATED PROTEIN KINASE (RAS/RAF/MEK/ERK) PATHWAY

The MAPK pathway is a highly conserved signaling cascade involved in various cellular functions including cell proliferation, differentiation, and migration. This pathway can be activated by the stimulation of upstream signaling molecules including growth factor receptors and G protein-coupled receptors (Wellbrock et al., 2004a; Gray-Schopfer et al., 2007). The aberrant activation of the classical MAPK pathway with extracellular signal-regulated kinase (ERK) as the terminal kinase is a frequent event in human cancer and is often the result of activating mutations in the oncogenes; *BRAF* (7%; Davies et al., 2002) and *RAS* (15–30%; Bos, 1989) based on analyses of all cancer types. It is interesting to note that mutations of *RAS* and *RAF* are mutually exclusive in associated malignancies including melanoma (Brose et al., 2002).

## RAS

The RAS proteins (H, K, and N-RAS) are small GTPases localized on the inner leaflet of the plasma membrane where they serve as critical mediators of cell growth, proliferation and differentiation (Trahey and McCormick, 1987; Lowy and Willumsen, 1993). RAS activity is controlled through cycling between a guanosine diphosphate (GDP)-bound state (inactive) and a guanosine triphosphate (GTP)-bound state (active; Downward, 1996; Scheffzek et al., 1997). The cycling between GDP- and GTP-bound state is partially controlled by the intrinsic GTPase activity of RAS, the activity of GTPase-activating proteins (GAPs) which promote the formation of inactive RAS–GDP complexes, and guanine-nucleotide exchange factors (GEFs) that accelerate the formation of RAS–GTP complexes (Cales et al., 1988; Herrmann et al., 1996). Mutations in the *RAS* genes abolish the intrinsic GTPase activities of these molecules and also reduce sensitivity to GAPs by preventing the dissociation of GTP (Trahey and McCormick, 1987; Scheffzek et al., 1997; Wittinghofer et al., 1997). GTP-bound RAS is able to activate its effector molecules such as RAF (Marais et al., 1995) and phosphatidylinositol-3-OH kinase (PI3K; Rodriguez-Viciana et al., 1994), and it is through the activation of these effectors that *RAS* is able to regulate proliferation, survival, and processes linked to tumorigenic cell transformation. The MAPK pathway can also be stimulated by phosphorylation of RAF by RAS (Marais et al., 1995; Weber et al., 2001), which in turn phosphorylates and activates MAPK kinases 1 and 2 (MEK1 and MEK2), which then phosphorylate and activate ERK1 and ERK2 (Rubinfeld and Seger, 2004; Rapp et al., 2006). Activated ERK1/2 phosphorylates numerous transcription factors that control gene expression such as *ELK1* (Babu et al., 2000), *FOS* (Monje et al., 2005), and *c-JUN* (Lopez-Bergami et al., 2007). RAS can also activate the PI3K/AKT signaling cascade through its interactions with the p110 catalytic subunit of PI3K (Rodriguez-Viciana et al., 1994; Pacold et al., 2000) leading to activation, translocation to the membrane, and conformational changes of the lipid kinase. PI3K phosphorylates phosphatidylinositol 4,5-bisphosphate [PtdIns(4,5)P_2_] to produce phosphatidylinositol 3,4,5-trisphosphate [PtdIns(3,4,5)P_3_], a second messenger that binds to a large number of proteins such as AKT/protein kinase B (PKB; Haslam et al., 1993; Datta et al., 1995; Franke et al., 1995) through pleckstrin homology domains. AKT is a modulator of oncogenic transformation (Mirza et al., 2000), cell survival (Edinger and Thompson, 2002), apoptosis (Cheung et al., 2008), cell cycle progression (Liang et al., 2002), and glycogen synthesis (Cross et al., 1995).

*N-RAS* is the most commonly mutated *RAS* isoform in human melanoma and melanocytic nevi (Der et al., 1986; Trahey and McCormick, 1987; Trahey et al., 1987). Mutational analyses have shown that ∼56% of congenital nevi exhibit *RAS* mutations in comparison to 33% of primary and 26% of metastatic melanomas (Albino et al., 1989; Jafari et al., 1995; Demunter et al., 2001). Activating *RAS* mutations are associated with sun and UV exposure and are more common in tumors under continuous UV exposure (56%) than tumors from intermittently or non-sun-exposed sites (21%; Ball et al., 1994; Jafari et al., 1995; van Elsas et al., 1996). The most frequent observed mutations are in codons 12, 13, and 61 and they lead to the loss of the intrinsic GTPase activity of RAS resulting in constitutive signaling and activation of downstream cascades (Der et al., 1986; Trahey and McCormick, 1987; Trahey et al., 1987). This improper signaling has been shown to promote aberrant cell proliferation (Dumaz et al., 2006), metastasis (Ackermann et al., 2005), inhibition of apoptosis (Kodaki et al., 1994; Eskandarpour et al., 2005), and chemoresistance (Kodaki et al., 1994; Rodriguez-Viciana et al., 1994).

Activating mutations of *K-RAS* in melanoma appear to be an extremely rare event occurring in only 2% of cases, with the most common missense mutation found in codon 12 (Shukla et al., 1989; Milagre et al., 2010). This mutation has been shown to induce anchorage-independent growth in melanocytes transformed with *K-RAS*^*G12V*^; however, it is less tumorigenic compared to cells transformed with *N-RAS*^*G12V*^ indicating that *K-RAS* may be a weaker oncogene than *N-RAS* in melanocytes (Whitwam et al., 2007). *H-RAS* mutations are also rare, detected only in 1% of melanomas (Milagre et al., 2010), especially sporadic melanomas and Spitz nevi likely from amplification of its genomic locus on chromosome 11p and oncogenic point mutations (Bastian et al., 2000). In animal models, tumorigenicity of mutant *H-Ras*^*G12V*^ has been shown to be enhanced in mice with deletions in *p16Ink4a* (Chin et al., 1997, 1999), mutation of *p53* (Bardeesy et al., 2001), or UV exposure (Hacker et al., 2005).

Given the role that RAS plays in cancer, various therapeutic strategies for targeting this oncogenic protein have emerged. Most challenging however, is the search for small molecule inhibitors that can directly target RAS through binding to active sites or binding pockets (Gysin et al., 2011). Several small molecule inhibitors that can suppress RAS activation by preventing guanine exchange through inhibition of RAS–GEF interactions have been identified (Taveras et al., 1997; Colombo et al., 2004; Peri et al., 2005). These small molecules bind to a cleft on the switch 2 region (residues 60–76) but their therapeutic potential is unknown. Inhibitors that target post-translational modifications of RAS have also been explored for therapeutic purposes. The attachment of a farnesyl isoprenoid group to RAS proteins is required for localization to the plasma membrane and activity (Kohl et al., 1995). Several farnesyltransferase inhibitors have been identified through rational design strategies (Dinsmore and Bell, 2003) and compound library screens (Sebti and Hamilton, 2000). These inhibitors have been shown to suppress the activity of mutated, constitutively active RAS in vitro (Kohl et al., 1995; Sebti and Hamilton, 2000) and tumor growth in vivo (End et al., 2001; Gunning et al., 2003). Despite these promising results, clinical validation of several of these inhibitors did not show objective responses in most solid tumors (Sharma et al., 2002). In melanoma, a phase II clinical trial of the farnesyltransferase inhibitor, R115777 (tipifarnib) as a single agent did not show any benefit (Gajewski et al., 2006). Furthermore, in a recently completed trial, tipifarnib in combination with sorafenib or temsirolimus did not show any activity to justify continued use (Margolin et al., 2012). Failures of farnesyltransferase inhibitors in vivo and in clinical trials have been attributed to RAS prenylation and reactivation via geranylgeranyl transferase type 1 (Britten et al., 2001; Lobell et al., 2001). The specificity of R115777 is to the rarely mutated H-RAS, instead of the more frequently mutated N-RAS or K-RAS, and has also been speculated to be a major cause of the reduction in efficacy (James et al., 1996; Baines et al., 2011). Success in targeting melanomas with RAS mutations may be achieved by inhibiting RAS effector pathways through combined targeting of BRAF, MEK, and PI3K/AKT/mammalian target of rapamycin (mTOR) due to the integral role of these effectors in RAS driven transformation as well as the availability of clinically tested small molecule inhibitors (Davies et al., 2007; Engelman et al., 2008; Fasolo and Sessa, 2008; Lee et al., 2010; Gysin et al., 2011).

## BRAF

BRAF is a serine/threonine kinase, a component of the MAPK pathway downstream of RAS and when activated, triggers phosphorylation of MEK (Johnson and Lapadat, 2002). Mutations in *BRAF* are prevalent in human cancers (7%) with the highest incidences found in malignant melanoma (27–70%), papillary thyroid cancer (36–53%), colorectal cancer (5–22%), and serous ovarian cancer (30%; Davies et al., 2002; Kumar et al., 2003; Pollock et al., 2003a; Young et al., 2005). Of the over 40 *BRAF* activating mutations identified, the *BRAF*^*V600E*^ mutation is the most common, and accounts for 92% of *BRAF* mutations in sporadic melanomas and 82% of benign nevi, implying that it might be involved in the progression from a benign to a cancerous state (Davies et al., 2002; Kumar et al., 2003; Pollock et al., 2003a). A single-base missense transversion (T to A at nucleotide 1,799) changes valine to glutamic acid in codon 600 (V600E) of exon 15, and results in constitutive activation of the RAF kinase (Davies et al., 2002; Garnett and Marais, 2004; Wan et al., 2004). Given the presence of the *BRAF*^*V600E*^ mutation in benign melanocytic nevi (Pollock et al., 2003a), pre-malignant colon polyps and early stage colorectal cancer (Yuen et al., 2002; Ikehara et al., 2005), the oncogenic potential of mutated BRAF has been under investigation. *BRAF*^*V600E*^ was shown to transform NIH3T3 fibroblasts and mouse melanocytes resulting in increased proliferation *in vitro*, stimulation of ERK and tumorigenesis *in vivo* (Houben et al., 2004; Ikenoue et al., 2004; Wan et al., 2004; Wellbrock et al., 2004a). Interestingly, benign melanocytic nevi with *BRAF* mutations exhibit growth arrest characteristics including the expression of the senescence marker, β-galactosidase (Michaloglou et al., 2005; Gray-Schopfer et al., 2006; Dhomen et al., 2009). This might suggest that other mutations are required to drive oncogenesis in nevi, which is supported by studies such as those showing that loss of *p53* results in the progression to melanoma (Patton et al., 2005). However, it is still possible that the benign nevi with mutated *BRAF* can escape the oncogene-induced senescence and become melanomas, which might explain the high percentage of this mutation in sporadic melanoma (Wellbrock et al., 2004b; Dhomen et al., 2009). The effects of other less frequent observed BRAF mutations have also been investigated. Among melanomas with mutated BRAF, the *BRAF*^*V600K*^ mutation is observed in 12% of cases while *BRAF*^*V600R*^ and *BRAF*^*V600D*^ are each observed at a frequency of ∼5% (Lovly et al., 2012). These mutations, similar to BRAF**^*V600E*^ result in an increase in BRAF kinase activity and increased MEK and ERK phosphorylation (Wan et al., 2004).

The high prevalence of the *BRAF*^*V600E*^ mutation in melanoma has made it a popular target in drug development. Small kinase inhibitors have yielded mixed results with some showing greater efficacy than others. Sorafenib (Nexavar, Bay 43-9006), was initially produced as a specific inhibitor of *CRAF* and was found to also have inhibitory activity toward *BRAF* (Lyons et al., 2001; Wilhelm et al., 2004). Further investigation showed that sorafenib not only inhibited wild-type BRAF, but mutant BRAF as well. Additionally, it also asserts inhibitory activity toward various receptor tyrosine kinases critical in cancerous processes including vascular endothelial growth factor receptor (VEGFR) 1/2/3, platelet-derived growth factor receptor β (PDGFR-β), fibroblast growth factor receptor 1 (FGFR-1), c-KIT, FLT-3, and RET (Wilhelm et al., 2004; Carlomagno et al., 2006; Lierman et al., 2006; Chang et al., 2007). Various studies have shown the potential of sorafenib in inhibiting the growth of a host of malignancies including melanoma, leukemia, hepatocellular carcinoma, esophageal carcinoma *in vitro* and *in vivo* (Wilhelm et al., 2004; Sharma et al., 2005), and is successfully utilized in the treatment of renal cell carcinoma (Escudier et al., 2009). Single agent sorafenib for melanoma treatment has been largely unsuccessful, with efficacy improved when used in conjunction with chemotherapy or adjuvant immunotherapy (Eisen et al., 2006; McDermott et al., 2008; Amaravadi et al., 2009; Augustine et al., 2010; Ott et al., 2010; Egberts et al., 2011).

Small molecule inhibitors with greater specificity to mutant *BRAF*^*V600E*^ than the wild-type protein have been developed. SB590885 (GlaxoSmithKline, Collegeville, PA, USA) was shown to have 100-fold more activity than sorafenib in inhibiting *BRAF* activity (King et al., 2006). Sorafenib stabilizes the inactive conformation of the kinase while SB590885 stabilizes the active *BRAF* conformation, which explains the difference in activity and might make SB590885 a better candidate for clinical development (King et al., 2006). Vemurafenib (PLX4720/RG7204), a novel *BRAF* inhibitor with high specificity to *BRAF*^*V600E*^ has potent cytotoxicity against melanoma cells *in vitro* and *in vivo* and clinically has improved survival of melanoma patients (Tsai et al., 2008; Yang et al., 2010; Chapman et al., 2011; Young et al., 2012). It also appears that similar to the BRAF^V600E^ mutations, the BRAF^V600D^, BRAF^V600K^, and BRAF^V600R^ mutations are also responsive to inhibition by vemurafenib in pre-clinical trials (Rubinstein et al., 2010; Yang et al., 2010). In clinical trials, BRAF^V600K^ and BRAF^V600E^ both show better responses to the MEK inhibitor, trametinib compared to dacarbazine therapy and also when compared to patients with wild-type BRAF tumors (Flaherty et al., 2012).

During a phase I clinical trial of vemurafenib, 81% of patients with *BRAF*^*V600E*^ mutations demonstrated significant shrinkage of liver, bowel, and bone metastases and progression-free survival of 7 months (Flaherty et al., 2010). The follow-up phase II trial showed a response rate of 52% (Bollag et al., 2010). Meanwhile, 48% of patients showed a partial response in a phase III trial, with 0.9% complete responses observed (Chapman et al., 2011). The limiting factor in patient treatment with vemurafenib appears to be innate and acquired resistance. Furthermore, it appears that there are alterations in signaling after BRAF inhibitor exposure that may promote cell growth indicating that meticulous selection of treatment candidates is necessary. This is especially important because some patients treated with vemurafenib present with dermatological side effects that include keratoacanthomas and squamous cell carcinomas (Oberholzer et al., 2012; Su et al., 2012). Reports indicate that BRAF inhibitors induce ERK signaling and increase growth in wild-type BRAF cells (Heidorn et al., 2010; Poulikakos et al., 2010). Further studies have shown that exposure to BRAF inhibitors results in increased binding of BRAF to CRAF, especially in RAS mutant cells leading to hyperactivation of CRAF, and elevated ERK signaling (Hatzivassiliou et al., 2010). Subsequent analysis showed that this increase was as a result of transactivation of RAF dimers by BRAF inhibitors (Hatzivassiliou et al., 2010; Poulikakos et al., 2010). The binding of a BRAF inhibitor to one protomer within a RAF dimer was found to result in loss of the catalytic activity of the inhibitor-bound RAF and transactivation of the other protomer. This transactivation of RAF homo- and heterodimers is likely responsible for induction of MEK/ERK phosphorylation by RAF inhibitors in cells with wild-type BRAF. The keratoacanthomas and squamous cell carcinomas observed in vemurafenib treated patients show a high rate of RAS mutations and increased ERK signaling despite having the BRAF^V600E^ mutation and treatment with the drug suggesting that the RAS mutations may pre-dispose the patients to these dermal lesions. Acquired resistance mechanisms are also under investigation. Recently, it has been shown that innate resistance to vemurafenib can be attributed to the secretion of hepatocyte growth factor (HGF) by the tumor micro-environment (Straussman et al., 2012). This results in the activation of the HGF receptor, MET, which can reactivate the MAPK and PI3K/AKT pathways (Straussman et al., 2012). Other mechanisms of acquired resistance have also been attributed to reactivation of the MAPK and PI3/AKT pathways via development of *N-RAS* mutations (Nazarian et al., 2010), activation of AKT (Shao and Aplin, 2010), up-regulation and enhanced activation of the receptor tyrosine kinases PDGFR-β (Nazarian et al., 2010), COT/MAP3K8 (Johannessen et al., 2010), insulin-like growth factor 1 receptor (IGF-1R), FGFR3 (Yadav et al., 2012), emergence of an aberrantly spliced BRAF variant [p61BRAF(V600E); Poulikakos et al., 2011] and increases in *BRAF*^*V600E*^ copy number (Shi et al., 2012). Other *BRAF* inhibitors such as GDC0879 (Hoeflich et al., 2009; Wong et al., 2009) and GSK2118436/dabrafenib (Anforth et al., 2012; Hauschild et al., 2012) are currently in the development and testing phase to determine their efficacy in melanoma treatment. In clinical testing, dabrafenib was shown to improve progression-free survival with durable responses at 6 months (Falchook et al., 2012b; Hauschild et al., 2012).

To circumvent the innate and acquired resistance problem, combinations of *BRAF* inhibitors with inhibitors of other kinases and pathways that promote melanoma growth are being investigated. Co-inhibition of BRAF^V600E^ with MEK (Flaherty et al., 2012; Shi et al., 2012), PI3K/mTOR (Greger et al., 2012), metabotropic glutamate receptor 1 (Lee et al., 2011; Mehnert et al., 2012), histone deacetylases (Lai et al., 2012), Hsp90 (Catalanotti and Solit, 2012), and cytotoxic T lymphocyte antigen 4 (CTLA-4; Weber et al., 2012) are actively being pursued. The combination of vemurafenib and the CTLA-4 blocker, ipilimumab, is thought to be especially promising as evidence suggests that BRAF inhibitors and immunotherapy may act synergistically (Ascierto et al., 2012). Pre-clinical studies indicate that exposure to high concentrations of PLX4720 does not affect the viability and function of lymphocytes (Comin-Anduix et al., 2010). Furthermore, other studies have shown that PLX4720 treated cells become better targets for immunotherapy due to increased expression of melanocyte differentiation antigens which confer enhanced antigen-specific recognition by CTLs (Boni et al., 2010).

## MEK1/2

MEK1/2 are kinases that phosphorylate tyrosine and threonine residues on ERK1/2 kinases (Roskoski, 2012). MEK mutations are rare in human cancers with minimal mutated cases detected in lung cancer (Marks et al., 2008; Sasaki et al., 2010) and ovarian cancer (Estep et al., 2007). Analyses of human melanoma tumors have also shown a low incidence (3–8%) of somatic mutations in MEK (Murugan et al., 2009; Nikolaev et al., 2012). Regardless, MEK inhibitors have emerged as an effective strategy to target drug resistant *BRAF*^*V600E*^ melanomas in patients with or without previous exposure to BRAF inhibitors (Gilmartin et al., 2011; Wagle et al., 2011). Trametinib (Falchook et al., 2012a; Flaherty et al., 2012) and selumetinib (Boers-Sonderen et al., 2012) have emerged as potent MEK inhibitors. Pre-clinical studies show that cells with mutated BRAF are sensitized to AZD-6244/selumetinib (Prickett et al., 2011; Dahlman et al., 2012), TAK-733 (Dahlman et al., 2012). Furthermore, clinical studies have also shown that MEK inhibitors increase sensitization to BRAF inhibition with improved survival achieved in patients treated with combination MEK and BRAF inhibitors compared to either drug alone (Flaherty et al., 2012).

## PI3K/AKT PATHWAY

Activation of the PI3/AKT pathway is one of the most frequent events in cancer. This pathway is a critical player not only in normal physiological processes but also in tumorigenic development through the positive regulation of G1/S phase progression, inhibition of apoptotic cell death, and increased survival (Cully et al., 2006; Jiang and Liu, 2008; Yuan and Cantley, 2008). When activated by any one of a variety of mechanisms including activated receptor tyrosine kinases (Domchek et al., 1992), interactions with growth factor receptor-bound protein 2 (GRB2) adaptor protein (Pawson, 2004), or RAS (Kodaki et al., 1994; Rodriguez-Viciana et al., 1994; Chan et al., 2002), the second messenger lipid PtdIns(3,4,5)P_3_ is generated. PtdIns(3,4,5)P_3_ in turn recruits both phosphatidylinositol-dependent kinase 1 (PDK1) and AKT/PKB to the membrane where PDK1 phosphorylates and activates AKT/PKB and indirectly activates the mTOR (Hay and Sonenberg, 2004; Sarbassov et al., 2005). Activated AKT has multiple functions including increased oncogenic transformation, survival, proliferation, insulin metabolism, and cell cycle regulation (Stambolic et al., 1998; Mirza et al., 2000; Shin et al., 2002, 2010; Stahl et al., 2004). AKT can also directly phosphorylate mTOR through phosphorylation (and inactivation) of tuberous sclerosis complex 2 (TSC2), an inhibitor of mTOR (Ma et al., 2005). The activation of mTOR has been shown to be involved in regulation of glucose availability in the cell and tumorigenesis (Kim et al., 2003; Sarbassov et al., 2005). Dysregulation of the PI3K/AKT pathway in cancer can occur as result of mutations in the gene encoding the p110 catalytic subunit of PI3K, PI3KCA subunit (Samuels et al., 2004), loss of the tumor suppressor PTEN, a negative regulator of PI3K/AKT pathway (Li et al., 1997) or molecular alterations in AKT (Staal, 1987; Bellacosa et al., 1995; Cheung et al., 2008). In melanoma, PTEN loss and AKT amplification are common events and have been well documented.

## PHOSPHATASE AND TENSIN HOMOLOG

The tumor suppressor on chromosome 10, *PTEN* (deleted on chromosome 10) acts as a negative regulator of the phosphatidylinositol 3-kinase (PI3K) signaling pathway and has been implicated in a multitude of cancers. PtdIns(3,4,5)P_3_ is a key cell signaling molecule catalyzed from PtdIns(4,5)P_2_ by PI3K (Salmena et al., 2008). PTEN hydrolyzes the 3-phosphate on PtdIns(3,4,5)P_3_ to generate PIP_2_, and thereby negatively regulates PtdIns(3,4,5)P_3_-mediated downstream signaling (Stambolic et al., 1998; Carracedo and Pandolfi, 2008). Upon *PTEN* loss, PtdIns(3,4,5)P_3_ accumulates and promotes the recruitment of a subset of proteins that contain a pleckstrin homology domain to cellular membranes, including the serine/threonine kinases AKT1, AKT2, AKT3, and PDK1 (Stambolic et al., 1998). Deletion, mutation, or inactivation of *PTEN* results in aberrant activation of PI3K pathway effectors (Stambolic et al., 1998; Suzuki et al., 1998). Various alterations in *PTEN* have been identified in melanoma including allelic loss in 20% of melanomas, altered expression in 40% of tumors and hemizygous deletions and inactivation in 57–60% of melanoma cell lines (Pollock et al., 2002; Goel et al., 2006; Li and Ross, 2007; Yin and Shen, 2008). Ectopic expression of *PTEN* in melanoma cells lacking functional protein has been shown to inhibit AKT phosphorylation, increase apoptosis, and decrease cell proliferation (Stewart et al., 2002). siRNA knockdown of wild-type PTEN has been shown to result in increased phosphorylation of AKT3 and radial growth reinforcing its involvement in melanoma pathogenesis (Stahl et al., 2004). The lack of functional *PTEN* also appears to regulate cell survival by increasing *BCL-2* expression and promoting insensitivity to chemotherapeutic agents (Wu et al., 2003; Stahl et al., 2004; Madhunapantula et al., 2007). In melanoma, the loss of *PTEN* is thought to occur early in melanomagenesis as shown in primary lesions harboring loss of one allele of *PTEN*, or *PTEN* haplo-insufficiency due to the loss of the entire chromosome 10 (Parmiter and Nowell, 1988; Bastian et al., 1998; Wu et al., 2003). Several studies have shown that *PTEN* loss can interact with other melanoma mutations. Bosenberg’s group elegantly demonstrated that in a genetically modified mutated *BRAF* transgenic mouse model, the deletion of a functional *PTEN* can drive the development of malignant melanoma (Dankort et al., 2009). Furthermore, other studies have identified functional redundancy between *PTEN* loss and *RAS* mutation and have shown that these two genes are mutually exclusive in melanoma development due to redundant activation of the PI3K/AKT pathways (Tsao et al., 2000, 2004). *De novo* *Ras* mutations have been observed in a mouse model of *Pten*^*+/+*^ mice while *Pten*^+/−^ melanomas showed a decreased incidence of *Ras* mutations, while *Pten*^−/−^ mice completely lacked Ras mutations (Mao et al., 2004). Furthermore, Tsao et al. (2000) observed similar results in human melanoma cell lines where cells with *PTEN loss* lacked *RAS* mutations. Similarly, a mouse model of *Tyr-H-RAS*^*V21G*^ink4a/Arf^−/−^ in a *Pten*^+/+^ or *Pten*^+/−^ background showed that inactivation of one copy of *Pten* led to earlier onset of melanoma whereas mice without activated *Ras* in the *Pten*^+/−^Ink4aArf^−/−^ background did not give rise to animals with melanoma (Nogueira et al., 2010). Taken together, these studies suggest that activation of *Ras* and loss of *Pten* cooperates in a subset of melanomas. However, exceptions in the reciprocity of *NRAS* mutations and *PTEN* loss have been noted. In the study by Tsao et al. (2000), they found that one cell line in their cohort had concurrent loss of *PTEN* with an *NRAS* mutation. Similarly, Nogueira et al. (2010) found that ∼14% of the human melanomas they analyzed had an *NRAS* mutation in addition to loss of *PTEN*. It is possible that a small population that harbors both *RAS* and *PTEN* mutations has escaped from signaling through the PI3K pathway and instead its tumorigenic properties are driven by the MAPK pathway.

## AKT

Phosphatidylinositol (3,4,5)-triphosphate directly binds to PDK1 which can phosphorylate and activate AKT (Alessi et al., 1997; Currie et al., 1997). *AKT* has three isoforms; *AKT1*, *AKT2*, and *AKT3* with each encoded for by different genes which share a high degree of structural similarities (Staal, 1987; Nakatani et al., 1999). Upon PtdIns(3,4,5)P_3_ binding, PDK1 induces AKT kinase activity 30-fold by phosphorylating it on the catalytic domain on residue threonine 308, or through phosphorylation on the carboxy-terminal hydrophobic motif on serine 473 by PDK2 (Alessi et al., 1997; Toker and Newton, 2000). Phosphorylation of both sites has been shown to be essential for maximal activation of AKT (Alessi et al., 1996). These activated AKT serine/threonine kinases, in turn are thought to phosphorylate ∼9,000 proteins with the minimal recognition sequence: R-X-R-X-X-S/T in both the cytoplasm and the nucleus (Lawlor and Alessi, 2001). These proteins are involved in regulating the cell cycle, preventing apoptosis, and triggering cellular growth (Manning and Cantley, 2007).

Expression of these three *AKT* isoforms has been shown to be differential among tissues. *AKT1* is ubiquitously expressed in most organs and tissues at high levels; *AKT2* expression is preferentially elevated in insulin-sensitive tissue such as the liver, muscle, and adipose tissue while *AKT3* is predominantly expressed in the brain and testis (Dong et al., 1999; Zinda et al., 2001; Franke, 2008); expression however does not always imply activation (Stahl et al., 2004). All three isoforms of *AKT* have been linked to cancers of the stomach, breast, pancreas, and ovary (Staal, 1987; Cheng et al., 1992, 1996; Bellacosa et al., 1995). Dysplastic nevi and melanomas display increased *AKT* phosphorylation in contrast to normal or slightly dysplastic nevi (Dhawan et al., 2002). *AKT2* and *AKT3* have emerged as the predominant forms that are dysregulated in melanoma. Activated AKT3 has been detected in 43–60% of sporadic metastatic melanoma when compared to normal melanocytes, an observation attributed to increased copy number of the *AKT3* gene (Stahl et al., 2004). Additionally, levels of phosphorylated AKT3 were found to correlate with melanoma progression suggesting that AKT3 might have a role in the aggressiveness of melanomas (Stahl et al., 2004). In addition to the increase in copy number that leads to improper *AKT3* activation, loss of *PTEN* has also been shown to contribute to *AKT3* up-regulation. siRNA knockdown of *PTEN* led to enhanced AKT3 phosphorylation in both melanocytes and human melanoma cells (Stahl et al., 2004). siRNA-mediated down-regulation of AKT3 conversely resulted in a decrease in cell survival and tumor growth (Stahl et al., 2004; Tran et al., 2008). AKT3 has also been shown to participate in resistance to BRAF inhibitors and suppression of AKT3 may lead to increased clinical responses with BRAF inhibitors (Shao and Aplin, 2010). *AKT2* over-activation has also been identified in melanoma, breast, and ovarian cancer (Arboleda et al., 2003; Yuan et al., 2003; Nogueira et al., 2010; Shin et al., 2010). Expression of *AKT2* in melanoma has been established in several different models of melanoma; a mutant *Ras* background (Nogueira et al., 2010) and one with ectopic expression of metabotropic glutamate receptor 1 (*Grm1*; Shin et al., 2010). In the metabotropic glutamate receptor model (Pollock et al., 2003b; Namkoong et al., 2007), examination of primary, nodal and in-transit metastasis yielded AKT2 and not AKT3 as the predominant activated isoform. In subsequent studies, Akt was shown to be a downstream target of Grm1 (Shin et al., 2010). Modulation of Akt2 expression levels in an inducible siRNA system lead to growth suppression *in vitro* and *in vivo* (Shin et al., 2010). Furthermore, siRNA knockdown of GRM1 in human melanoma cell also resulted in a decrease in AKT2 phosphorylation corroborating that AKT2 is a downstream target of GRM1 (Wangari-Talbot et al., 2012). Nogueira et al. (2010) have also shown that *PTEN* loss in a mutant *RAS* background can result in the selective activation of *AKT2*. This up-regulation of AKT2 was found to contribute to the increase in cell transformation, invasiveness of melanoma cells and a reduction in E-cadherin expression. In addition, using a complementary genetic approach, a dominant negative mutant of AKT2 led to a decrease in the invasiveness of the melanoma cells (Nogueira et al., 2010). Regardless of which AKT isoform is involved in melanoma, the PI3K/AKT pathway is an important therapeutic target in melanoma.

Several studies have pointed to the potential use of PI3K/AKT inhibitors in suppressing tumor growth *in vitro*, *in vivo* as well as in chemo-sensitization (Brognard et al., 2001; Stassi et al., 2005; Sinnberg et al., 2009; Hirai et al., 2010; Isosaki et al., 2011). PI3K inhibition by the irreversible inhibitor wortmannin or LY294002, can block AKT activation as well as compensatory mechanisms and has been used widely in mechanistic studies to dissect the mode of action of this pathway (Vlahos et al., 1994; Wymann et al., 1996; Garcia-Echeverria and Sellers, 2008). These two compounds however have pharmaceutical limitations such as off-target activities that prevent them from transitioning from the bench to the clinic (Bain et al., 2003; Knight and Shokat, 2007). Based on the wortmannin model, compounds with fewer limitations such as PWT-458 and PX-866 have been developed but neither of them have entered clinical trials yet (Garcia-Echeverria and Sellers, 2008). ZSTK474 a novel potent PI3K inhibitor with anti-tumor efficacy is undergoing safety assessment in solid malignancies (Yaguchi et al., 2006). Other AKT inhibitors such as isoselenocyanates, API-2, SR13668, BI-69A11, GSK690693, and MK-2206 have been shown to have anti-tumor activity in suppressing tumor growth and are undergoing further testing (Forino et al., 2005; Karst et al., 2006; Rhodes et al., 2008; Sharma et al., 2009; Hirai et al., 2010). In a clinical trial however, treatment with the AKT inhibitor perifosine/keryx showed no objective responses in patients with metastatic melanoma and had significant gastrointestinal side effects (Ernst et al., 2005). AKT inhibitors however may be helpful in patients with *BRAF*^*V600E*^ melanomas as Akt activation has been shown to cooperate with the mutant B-Raf to promote progression and chemoresistance (Tran et al., 2008; Shao and Aplin, 2010). It is therefore not surprising that combinatorial therapies utilizing an AKT inhibitor such as MK-2206 and the MEK inhibitor, AZD-6244, in patients with relapsed *BRAF*^*V600E*^ positive melanomas (clinical trial NCT01510444) are in clinical testing. Another possibility in targeting the AKT pathway in melanoma is through inhibition of mTOR signaling using rapamycin or rapamycin analogs. These mTOR inhibitors show anti-tumor properties *in vitro*, *in vivo* and the ability to improve sensitivity to chemotherapeutic agents (Faivre et al., 2006; Sinnberg et al., 2009). Treatment of melanoma patients with the mTOR inhibitor sirolimus in combination with carboplatin and paclitaxel displayed significant tumor regression (Meier et al., 2009). Promising results have also been observed with another mTOR inhibitor, evolorimus (Hainsworth et al., 2010; Si et al., 2012).

## CDKN2A/p16^INK4A^/ARF

Familial melanomas account for 8–12% of diagnosed melanomas (Greene and Fraumeni, 1979; Fountain et al., 1992). Genetic studies in large melanoma-prone families have demonstrated that loss of heterozygosity or mutations at the p16 locus co-segregate with melanoma susceptibility in familial melanoma kindred (Hussussian et al., 1994; Kamb et al., 1994; Berwick et al., 2006). The 9p21 locus encodes two distinct proteins; p16INK4A and p19Arf in mouse/p14ARF in humans) and has been shown to undergo frequent recombination and deletions in both spontaneous and familial melanoma (Kamb et al., 1994; Quelle et al., 1995). Exon 1α and 1β of the *CDKN2A* gene are driven by two different promoters which results in two alternate transcripts that share exons 2 and 3. The 1α transcript encodes the p16INK4A protein while the 1β transcript encodes the p19Arf protein (Serrano et al., 1993; Quelle et al., 1995). p16INK4A is involved in the regulation of the cell cycle through its control of the RB-regulated G1–S transition (Serrano et al., 1993; DePinho, 1998; Sherr and Roberts, 1999), while p19Arf acts as a tumor suppressor by stabilizing and enhancing p53 levels through the blockade of MDM2-mediated p53 ubiquitination and degradation (Chen et al., 1998; Kamijo et al., 1998; Pomerantz et al., 1998; Zhang et al., 1998). Population-based studies have been performed in an attempt to elucidate the lifetime risk of developing melanoma in families with these mutations (Bishop et al., 2002; Berwick et al., 2006; Goldstein et al., 2007; Harland et al., 2008; Cust et al., 2011). A study based on 80 melanoma-prone families consisting of 402 melanoma patients and 713 non-affected family members from North America, Europe, and Australia was used by the Melanoma Genetics Consortium to calculate the lifetime projected risk of developing the disease in CDKN2A carriers (Bishop et al., 2002). By age 80, the projected risk of developing melanoma in North America was 76%, 91% in Australia, and 58% in Europe. Analysis of the same sample for comparative risks conferred by p16INK4A or p14ARF did not yield statistical significant differences in the melanoma risk between the two mutations (Bishop et al., 2002). Germ line *INK4A* mutations (Hussussian et al., 1994; Kamb et al., 1994), polymorphisms in the 5^′^ and 3^′^ untranslated regions (UTRs) that alter translation or regulate mRNA stability of *p16INK4A* and promoter mutations of *p16INK4A* are all genomic alterations that have also been identified in association with 9p21-linked familial melanoma (Liu et al., 1999; Kumar et al., 2001). Studies have shown that inactivation of *p16Ink4a* increased susceptibility to both spontaneous and carcinogen-induced melanoma (Krimpenfort et al., 2001; Sharpless et al., 2001). p16INK4A has also been reported to cooperate with other oncogenes to promote melanomagenesis (Serrano et al., 1993; Chin et al., 1997; Ackermann et al., 2005). The combination of *p16INK4a* deficiency with activated *H-Ras* (Serrano et al., 1993; Chin et al., 1997), *N-Ras* (Ackermann et al., 2005), and *K-Ras* (Monahan et al., 2010) in mouse models have been shown to promote highly penetrant melanomas with short latency. Recently, p16INK4A has also been shown to have a role in regulating cellular oxidative stress. In response to potential DNA oncogenic stress such as UV exposure, melanocytes were found to upregulate the expression of p16INK4A mediated by the p38 stress-activated protein kinase (SAPK) pathway (Naidu et al., 2009; Jenkins et al., 2011). In p16INK4A-deficient cells, an increase in intracellular reactive oxygen species (ROS), was noted even in the absence of exogenous oxidative stress with restoration of p16INK4A found to restore ROS levels to normal levels (Jenkins et al., 2011). Interestingly, regulation of ROS by p16INK4A was found to be independent of both its functions in cell cycle control as well as the retinoblastoma protein. Other studies have reported on possible roles of p16INK4A outside of its cell cycle control functions. For example, Becker et al. (2001) have shown that some p16INK4A mutants still retain their ability to bind CDK4. The precise mechanism through which p16INK4 regulates ROS remains elusive.

*p19Arf* controls the stability of the *p53* tumor suppressor whose activity is abrogated by point mutations in many tumors during carcinogenesis (Greenblatt et al., 1994; Hollstein et al., 1994). In melanoma, the pathological role of *p53* is highly controversial as primary and metastatic melanomas have been found to have low incidences of *p53* allelic loss or point mutations (Yang et al., 2001). However, cases of highly penetrant and aggressive melanomas involving *p53* inactivation in mouse models have been reported (Bradl et al., 1991). Bardeesy et al. (2001) have shown that a transgenic mouse model, *Tyr-RAS/Trp53*^+/−^, characterized by the loss of a *p53* allele but with retention of *p19Arf* develops melanoma. Interestingly, a *p19Arf* deficiency in the *Tyr-RAS;Ink4a/Arf*^−/−^ mouse model with functional *p53* was also found to develop melanoma (Chin et al., 1997). This illustrates a reciprocal role of *p53* inactivation and loss of *Arf* suggesting that they have related functions and that *Arf* may serve as a regulator of *p53* (Sharpless and Chin, 2003). Various therapeutic strategies for restoring wild-type p53 activity are under investigation. Small molecules that stabilize p53 in its active biological conformation and antibodies that bind the p53 carboxyl-terminus and restore its DNA binding function have been shown to have apoptotic and chemo-sensitization activity (Hupp et al., 1992, 1995). Additional strategies involve the reactivation of p53 through inhibition of MDM2 using small molecules such as nutlin (Vassilev, 2004; Vassilev et al., 2004). These strategies have had mixed results as CP-31398, a compound found to stabilize wild-type p53 and rescue mutant p53 was found not to increase chemosensitivity in human melanoma cells (Luu and Li, 2003). Recent studies have shown that *p53* dysregulation in melanoma can also occur due to the up-regulation of a negative regulator of *p53*, MDM4 in a significant proportion of stage I–IV melanomas (65%; Marine and Jochemsen, 2005). Targeting the MDM4–p53 pathway using the small peptide SAH-p53-8 that binds MDM4 and disrupts MDM4–p53 complexes was shown to result in tumor growth inhibition and sensitization to chemotherapeutics including BRAF inhibitors (Gembarska et al., 2012).

Although the insight obtained from studies on these pathways in melanoma has led to significant improvements in drug development, treatment, and patient survival, complete cure still remains elusive. This is driving cutting edge research into discovering novel drug targets that may lead to greater improvements in design of therapies. Genomic sequencing of tumor genomes and exomes has led to the identification of genes with unexpected roles in melanoma formation, progression, and resistance to therapy. In the next section, we will discuss some of the novel targets identified from next generation sequencing high throughput screens that allow the sequencing of random DNA fragments with large coverage of the cancer genomes. Various changes such as rearrangements, copy number variations, base substitutions, and small indels have been identified with sufficient coverage to identify most somatic mutations in an individual cancer genome (Pleasance et al., 2010).

## GENOMIC SEQUENCING OF MELANOMA

Whole genome sequencing has allowed the identification of mutational signatures in multiple tumor types including melanoma (Ley et al., 2008; Pleasance et al., 2010; Link et al., 2011; Puente et al., 2011; Welch et al., 2011). Pleasance et al. (2010) reported on the first comprehensive somatic mutation screen of melanoma performed in the COLO-829 melanoma cell line. A total of 33,345 somatic base substitutions, 292 of them in protein coding sequences were recognized. Two of these somatic substitutions were identified in *SPDEF*, an ETS transcription factor family, which has been associated with progression of breast and prostate cancer (Sood et al., 2007). Further sequencing of 48 additional melanoma biopsy samples confirmed the presence of these base pair substitutions as well as a third somatic mutation in *SPDEF*. A missense mutation was also identified in *UVRAG*, a putative tumor suppressor that complements the ultraviolet sensitivity of xeroderma pigmentosum group C cells and also has a role in autophagy (Kim et al., 2008). In addition, an 8- to 12-fold increase in copy number on chromosome 3p which contains four complete genes: *RARB*, *TOP2B*, *NGLY1*, and *KS* *(OXSM)* and a four- to sixfold increase on chromosome 15 containing *MKRN3* and *NDN* genes were noted. It is important to point out that this was the first instance that these amplified candidate genes were implicated in cancer development. This study also identified a high rate of C to T transitions in the tumor samples that have been reported to be signatures associated with UV exposure (Daya-Grosjean and Sarasin, 2005; Pfeifer et al., 2005), suggesting that UV-induced DNA damage could have resulted in the pathogenesis of COLO-829 melanoma cells (Pleasance et al., 2010).

Turajlic et al. (2012) also performed whole genome sequencing on primary acral melanoma and matched lymph node metastasis from the same patient. A total of 12,661 base substitutions were identified in the primary acral melanoma while 11,711 base substitutions were identified in the metastatic specimen. Several single nucleotide polymorphisms were identified in *IFNA16*, which is within the melanoma susceptibility locus on 9p21, *MSH2*, *APC*, and *MEN1* and novel variants of *BRCA1* and *ERCC2* with the later two genes involved in DNA repair. Genomic amplification of several chromosomal regions; 4q12, 11q13, 11q14, 17p11, and 20q11 as well as of the receptor tyrosine kinase gene, *KIT*, were detected in both primary and metastatic samples. Other additional findings were the common C to T transitions at the 3^′^ base of pyrimidine di-nucleotides (TpC or CpC) associated with UV exposure (Daya-Grosjean and Sarasin, 2005; Pfeifer et al., 2005) indicating that similar to cutaneous melanomas, acral melanomas are just as susceptible to UV-induced DNA damage that contributes to melanoma development (Turajlic et al., 2012). Another genomic screen of acral melanomas likewise showed a high prevalence of UV associated C to T transitions in tumor samples consistent with melanomas arising from chronic sun exposure (Berger et al., 2012). A significant chromosomal rearrangement was found in the *PREX2* locus, which encodes a PtdIns(3,4,5)P_3_ RAC exchange factor recently shown to interact with and modulate the function of *PTEN* (Fine et al., 2009). In addition to the nine somatic rearrangements detected near the *PREX2* locus, amplification of *PREX2* was also identified in the tumor samples. Sequencing of another tumor cohort in the evaluation of *PREX2* mutations found a 14% frequency in non-synonymous mutations. Functional significance was assessed using truncation mutants and non-synonymous point mutations of *PREX2*. In comparison to wild-type *PREX2*, the over-expressed mutants showed accelerated tumorigenicity suggesting that some melanoma cells may gain oncogenic activity through *PREX2* mutations (Berger et al., 2012).

Exome screenings are another mechanism being used to examine melanoma tumor mutations. Wei et al. (2011) performed exome sequencing on 14 matched pairs of normal and metastatic tumor DNAs from untreated individuals with melanoma and focused on genes altered in more than two tumor samples. The common *BRAF*^*V600E*^ mutation was detected in 7 out of the 14 samples, while 9 other genes harboring recurrent mutations were also identified. One of these genes, *TRRAP* encodes a transformation/transcription domain-associated protein and functions as a component of a multi-protein co-activator complex possessing histone acetyltransferase activity that is central to the transcriptional activity of *p53*, *c-MYC*, and *E2F1*. *TRRAP* had a recurring serine to phenylalanine mutation at amino acid residue 722 in 6 out of the 14 samples suggesting that this might be mutational hotspot in melanoma. The clustering of this mutation is similar to the clustering of activation mutations found in *BRAF*, *NRAS*, or *PIK3CA* in melanoma suggesting it might be an oncogene. To assess the consequences of these substitutions on melanoma cells, knockdown of mutated *TRRAP* in melanoma cells resulted in increased apoptosis suggesting that these *TRRAP* mutations might be essential in the survival of melanoma cells. This screen also uncovered mutations in *GRIN2A*, an ionotropic (*N*-methyl-D-aspartic acid, NMDA) glutamate receptor subunit ε-1 in 6 out of the initial 14 samples as well as in 25.2% of additional melanoma biopsies and cell lines analyzed. The number of C to T transitions observed in *GRIN2A* was also significantly higher than the number of the other nucleotide substitutions. Two mutational clusters, and three recurrent mutations were found in evolutionarily conserved domains which by SIFT analysis are predicted to have protein function (Wei et al., 2011). The identification of this glutamate receptor supports the data by Chen and colleagues who have shown that an aberrantly expressed metabotropic glutamate receptor (*Grm1*) can result in melanocytic transformation *in vitro* and tumorigenesis *in vivo* (Zhu et al., 1998; Pollock et al., 2003b). In addition, significant subsets of human melanoma tumors express the human form of the receptor, GRM1 (Namkoong et al., 2007; Lee et al., 2011). In two completed clinical trials, targeting the glutamatergic signaling mediated by *GRM1* expression led to mixed clinical responses, pointing to the need of a better understanding of glutamatergic signaling and melanoma (Yip et al., 2009; Mehnert et al., 2011, 2012). Activating mutations in another metabotropic glutamate receptor *GRM3*, was also identified in an exon capture screen of G protein-coupled receptors in melanoma (Prickett et al., 2011). The initial screen showed that *GRM3* had a 16.3% mutation rate with 18 non-synonymous mutations in 13 of 80 tumors while a screen of an additional tumor cohort of 57 samples detected a 15.7% mutation rate. Among the mutations detected in *GRM3*, the Glu870Lys mutation was identified in 4 samples suggesting that this is likely a mutational hotspot in this gene. Functional screens performed with cells transformed with mutated *GRM3* showed enhanced activation of MEK1/2, increased migration *in vitro* and pulmonary metastasis in xenograft models. Interestingly, it was also shown that cells with *GRM3* activation mutations are more responsive to treatment with the MEK inhibitor AZD-6244 than *GRM3* wild-type cells (Prickett et al., 2011). *GRM3* might turn out to be an important player in melanoma as an independent exome screen from the Halaban group also identified it as one of the genes with a high mutation burden in sun-exposed melanomas (Krauthammer et al., 2012). Furthermore, given the low success rates observed with MEK inhibitors, *GRM3* activating mutations could be a predictor of MEK inhibitor responsive tumors (Prickett et al., 2011).

Krauthammer et al. (2012) performed an exome sequencing of 147 primary and metastatic tumors which was a significantly bigger sample size than analyzed previously by other groups. Comparison of the 147 melanomas with matched samples revealed 23,888 missense mutations, 1,596 non-sense mutations, 399 splice-site variants, and 282 insertions/deletions. Comparative analysis of sun-exposed versus sun-shielded melanomas showed that sun-exposed melanomas found on the trunks, arms, legs, and head had a higher prevalence of somatic mutations compared to the sun-shielded acral, mucosal, and uveal melanomas. In addition, tumors from older patients were found to contain more mutations than those in younger people with the primary lesions of the older patients found in the head and neck, which is indicative of melanomas arising due as a result of chronic sun damage. Based on sun exposure and mutation burden, the investigators were able to classify the tumors into three distinct groups corresponding to the number of mutations present namely, high, medium, and low mutation count. These mutations likely originated in lesions from chronically exposed, intermittently sun-exposed and sun-shielded skin regions, respectively. Similar to other exome sequencing studies, a significant proportion of the single base pair mutations included C > T transversions associated with UV-induced DNA damage. Furthermore, they identified a motif, TTTCGT, enriched in sites where three or more mutations were found on sun-exposed skin suggesting a potential hotspot for the formation of cyclobutane pyrimidine dimers which are associated with lesions arising after UV exposure. Of the genes found to be frequently mutated, *BRAF* and *NRAS* featured prominently in lesions found on sun-exposed areas. Most interesting, a novel recurrent mutation was also identified in these sun-exposed melanomas. The recurrent mutation identified in seven of the tumor samples was a substitution of a proline for a serine at amino acid 29 in RAC1 (Ras-related C3 botulinum toxin substrate 1; RAC1^P29S^), a small Rho GTPase family protein with roles in proliferation, migration, and cytoskeletal rearrangements. Analysis of an additional set of 364 tumors detected the RAC1^P29S^ mutation in 20 of the samples (9.2%) and also in 4 out of 76 cell lines (5.3%) derived from sun-exposed tumors. There was no difference in the frequency of the mutation in primary versus metastatic tumors. Of note however, is the higher frequency in men (12.8%) versus women (2.4%) attributed to higher rates of UV exposure in men than women. In *in vitro* assays, RAC1^P29S^ was shown to be a gain of function mutation, 4.5-fold more active in its GTP-bound state compared to the wild-type protein. In transiently transfected cells, RAC1^P29S^ was shown to exhibit increased binding to the downstream effectors PAK1 and MLK3, enhance ERK phosphorylation, cell proliferation, and migration in comparison to the wild-type protein. In addition, it appears that *RAC1*^*P29S*^ frequently associates with the netrin 1 receptor, *DCC*, a tumor suppressor which can mediate signals that promote proliferation and migration. It is possible that *RAC1*^*P29S*^ and *DCC* loss cooperate in a manner similar to that of PTEN loss and mutations in BRAF or RAS in promoting melanoma tumor growth. In addition, they also found several mutated genes in sun-shielded melanomas. Mutations in *DYNC1I1* dynein, cytoplasmic 1, intermediate chain 1, which encodes a protein with roles in microtubule motor activity, progression through the spindle assembly checkpoint, and normal chromosome segregation were found in 3 of 17 acral melanomas. A second RAC1 mutation, due to a substitution in amino acid 65, Asp65Asn, was found also found in acral melanomas. In six uveal melanomas, mutations in BAP1 were also identified. Thus it appears that distinct mutational signatures exist in lesions depending on the amount of sun exposure and the resulting UV-induced DNA damage. Further, the newly identified RAC1^P29S^ may have therapeutic potential given its cancer-related signaling.

Chin and colleagues similarly reported on a whole exome sequencing study in which they examined paired tumor and normal DNA from 135 melanoma patients in a challenge to differentiate passenger mutations from driver mutations (Hodis et al., 2012). Over 83,000 mutations were identified, with most of them non-synonymous which may suggest that they are passenger mutations and not drivers. In this study, and similar to the previously discussed reports mutation signatures associated with UV exposure were highly predominant. Permutation based framework was used to identify non-silent mutations with predicted functional significance which identified eleven genes with high significant mutation burdens that included *BRAF*, *NRAS*, *TP53*, *PTEN*, *P16INK4A*, and *MAP2K*, as well as new candidates that included RAC1, *PPP6C*, *SNX31*, *TACC1*, and *STK19*. It is important to note that *RAC1* and *PPP6C* were also identified in the screen by Krauthammer et al. (2012). In this study, RAC1^P29S^ was also shown to have increased effector binding as well as increased association with GTP compared to the wild-type protein. In addition, they also identified MAP2K1 as a mutated gene in melanoma, with a recurrently mutated hotspot which confirmed a prior report (Nikolaev et al., 2012). It is important to note that despite converging on some of the same genes using different analysis methodology, there are disparities with genes identified in one screen and not identified in another which may be due to the filters applied for each analysis. Regardless, the permutation framework applied by Chin and colleagues for this analysis may be especially useful for screening bigger sample sizes (Hodis et al., 2012).

Whole exome sequencing is also been used to investigate acquired resistance resulting in drug relapse in patients treated with BRAF inhibitors such as vemurafenib (Shi et al., 2012). In a study by Shi et al. (2012), 20 sets of matched pre- and post-vemurafenib treatment biopsy samples were subjected to whole exome sequencing. An increase in *BRAF*^*V600E*^ copy number (2- to 14-fold) was noted in patients who initially responded then relapsed with disease progression. In addition, an increase in mutant *BRAF* to wild-type *BRAF* ratio was also noted in the patient samples that showed increased *BRAF*^*V600E*^ copy number suggesting the possible selection for the mutant genotype during the resistance acquisition process. This selection was confirmed in experiments performed in vemurafenib resistant human melanoma cell lines derived from *BRAF*^*V600E*^-vemurafenib responsive cells lines under continuous drug exposure. Furthermore, they showed that drug saturation of the mutant BRAF^*V600E*^ protein could be achieved by increasing the dose as copy number gain conferred resistance to a lower concentration (1 μM) but not a higher concentration (10 μM) implying that dose escalation of vemurafenib or other *BRAF* inhibitors might overcome the acquired resistance (Shi et al., 2012).

Genomic studies have played significant roles in improving treatment protocols for melanoma by expanding our ability to design targeted therapies. In addition, we have also gained insight on how to modify these therapies to achieve maximal results through different combination therapies. Monotherapies for melanoma have been shown to slow disease progression and also increase survival with varying success. Combination therapies have emerged as means to increase survival and long-term remissions. Importantly, it is now easier to predict whether a patient is likely to respond to a particular form of therapy due to the mutational signatures of their tumors. Next generation sequencing and other high throughput screens also continue to uncover genes with novel oncogenic properties in melanoma which open opportunities for drug design. Furthermore, algorithms and permutations may make the process of analyzing large samples and sorting mutations based on significance and potential functions a less complex. The clinical potential of some of these novel melanoma candidate genes, such as *GRM3* are already clear and given the speed at which modern science is advancing, we can speculate that the information gained from these sequencing studies will in the future be applied toward clinical medicine. Moreover, it is important to also take note of the not so surprising revelations of these sequencing projects especially as they relate to UV exposure and its role in DNA damage and melanoma formation. With an increase in sun seeking behavior and tanning, it is critical that this information is shared with the general public population in the hope that behavior modification will occur in order to reverse the rising incidence of melanoma.

## Conflict of Interest Statement

The authors declare that the research was conducted in the absence of any commercial or financial relationships that could be construed as a potential conflict of interest.
